# Mediterranean Diet and Airway Inflammation in School-Aged Children

**DOI:** 10.3390/children10081305

**Published:** 2023-07-29

**Authors:** Mónica Rodrigues, Francisca de Castro Mendes, Patrícia Padrão, Luís Delgado, Inês Paciência, Renata Barros, João Cavaleiro Rufo, Diana Silva, André Moreira, Pedro Moreira

**Affiliations:** 1 Faculty of Nutrition and Food Sciences, University of Porto, 4150-180 Porto, Portugal; carolina101997@hotmail.com (M.R.); renatabarros@fcna.up.pt (R.B.); andremoreira@med.up.pt (A.M.); pedromoreira@fcna.up.pt (P.M.); 2 Basic and Clinical Immunology, Department of Pathology, Faculty of Medicine, University of Porto, 4200-319 Porto, Portugal; francisca_castromendes@hotmail.com (F.d.C.M.); ldelgado@med.up.pt (L.D.); disolha@gmail.com (D.S.); 3 Epidemiology Research Unit, Laboratory for Integrative and Translational Research in Population Health, Institute of Public Health, University of Porto, 4050-600 Porto, Portugal; jcrufo@gmail.com; 4 Immuno-Allergology Department, Centro Hospitalar São João, 4200-319 Porto, Portugal; 5 Center for Health Technology and Services Research (CINTESIS@RISE) , Faculty of Medicine, University of Porto, 4200-319 Porto, Portugal; 6 Center for Environmental and Respiratory Health Research (CERH), Population Health, University of Oulu, 90014 Oulu, Finland; inespaciencia@gmail.com; 7 Biocenter Oulu, University of Oulu, 90014 Oulu, Finland

**Keywords:** airway inflammation, inflammation, Mediterranean diet, body mass index, obesity, childhood

## Abstract

There seems to exist an intricate relationship between airway inflammation, body mass index (BMI), and diet. The intake of specific foods or food groups has been suggested to suppress the oxidative stress and inflammatory processes that characterize airway inflammation, but little is known about dietary patterns and their complex interplay with BMI and airway inflammation. Therefore, this cross-sectional study aimed to explore the association between adherence to the Mediterranean diet (MD), a characteristic European diet, and levels of airway inflammation in school-aged children, taking into account their BMI. This cross-sectional analysis comprised 660 children: 49.1% females, 7–12 years old. Adherence to the MD was assessed through the alternate Mediterranean score (aMED). Higher scores represent a healthier diet (0–8). Airway inflammation was assessed measuring exhaled fractional nitric oxide (eNO). Two categories of BMI were considered: non-overweight/non-obese (*p* < 85th) and overweight/obese (*p* ≥ 85th). The associations between diet and airway inflammation were estimated using logistic regression models. Higher scores of the aMED were associated with decreased odds of having eNO ≥ 35 ppb, but only in non-overweight/non-obese children (OR = 0.77; 95% CI, 0.61–0.97). For overweight/obese children, the previous association was not significant (OR = 1.57, 95% CI, 0.88–2.79). Our findings suggest that adherence to the MD is associated with lower levels of airway inflammation among non-overweight/non-obese children.

## 1. Introduction

Chronic inflammation is linked to deleterious effects, such as damage to healthy tissues, as occurs in asthma and other respiratory diseases [1]. Several studies are nowadays focusing on biomarkers to detect inflammation to aid clinicians in the diagnosis and management of this condition [2]. One such biomarker is exhaled nitric oxide (eNO), which has gained prominence due to its clinical feasibility. Exhaled nitric oxide evaluation is especially relevant in children as it is a simple, non-invasive method to measure airway inflammation [1,3,4,5,6].

Exhaled nitric oxide serves as an indicator of inflammation in the airways, specifically highlighting the presence of eosinophilic inflammation [2,7]. NO is a reactive gas, functioning as a free radical, which is produced in the airways through the oxidation of l-arginine, converting it to l-citrulline [7]. NO is generated in the airways through the activity of two distinct enzymes: constitutive nitric oxide synthase (cNOS), responsible for producing small quantities of NO under normal conditions, and epithelial inducible NOS (iNOS), which becomes activated in response to inflammatory cytokines, leading to increased NO production [2,7].

The occurrence of inflammation in the lower airways can be attributed to a combination of several key factors, including genetic predisposition, environmental exposures, and possible alterations in the microbiota. These elements collectively contribute to the development of airway inflammation [8]. An imbalance in the adipose tissue as is found in obesity also leads to a pro-inflammatory environment both systemically and in the respiratory system [9]. Adipose tissue comprises an endocrine organ that produces hormones that may impact inflammation and metabolism. When fat accumulates in the body, it leads to higher levels of serum leptin and lower levels of adiponectin [10,11]. The higher production of leptin by adipose tissue can stimulate inflammation by triggering neutrophil chemotaxis, stimulating the generation of reactive oxygen species (ROS), activating natural killer cells and macrophages, and enhancing the production of T-helper-1 cytokines like interleukin (IL)-6 and Interferon-γ [10]. Conversely, adiponectin counteracts the effects of proinflammatory cytokines on endothelial and other cell types, such as tumor necrosis factor (TNF)-α and IL-6, while also being able to induce the expression of anti-inflammatory cytokines (IL-10 and IL-1 receptor antagonist) [12]. 

Another factor that affects airway inflammation implicates gut microbiota changes. Obesity has been correlated to a reduction in the variety of bacteria in the gut [13,14], and there is evidence that diet-induced weight loss promotes a decrease in the Firmicutes-to-Bacteroidetes ratio [15]. Bacteria belonging to the Firmicutes group produce a larger amount of butyrate, which has immunomodulatory and anti-inflammatory effects [16]. Fiber has the capability to influence the gut microbiota, resulting in the generation of short-chain fatty acids (SCFAs), such as butyrate [17]. Consuming a high-fat diet alters the composition of the gut microbiota, leading to an increase in invasive bacteria and a decrease in beneficial bacteria, along with reduced levels of SCFAs. This suggests a possible link to inflammation and immune response [18]. The alterations in microbiota could potentially contribute to airway inflammation by affecting the production of bacterial-derived or modified metabolites, including a potential decrease in the production of SCFAs [19]. 

Additionally, a lack of antioxidants due to limited consumption of fruits and vegetables, along with the consumption of foods high in saturated fats and adherence to a typical obesity-promoting diet, such as the Western-style diet, can elevate oxidative stress levels in the airways. This, in turn, leads to the production of ROS and subsequent damage to the lungs through various oxidative and inflammatory processes [20].

The majority of studies have primarily concentrated on examining the effects of specific components or nutrients in order to gain a deeper understanding of how diet influences respiratory health [18,21,22]. Nonetheless, attempts to improve health outcomes through interventions focused on supplementing individual components have shown disappointing results [23,24,25,26,27,28,29]. It is crucial to take into account the overall dietary pattern to comprehend the potential synergistic impacts of different food components [18,21,30].

Several researchers have attempted to identify indexes and dietary patterns that characterize the complete diet and that are associated with health [21,22,31]. We previously observed that diet, including dietary acid loads, dietary diversity, and diet quality, is associated with asthma-related mRNAs [32], airway inflammation and asthma [33], and markers of the exhaled breath condensate [34], respectively. Additionally, we also observed that diet quality is associated with asthma and with airway inflammation [35]. Nonetheless, in our last-mentioned study [35], we used the Healthy Eating Index-2015 for diet quality assessment; this index focuses on the dietary recommendations for Americans, so it may not be adequate to characterize the diet of the European population. The Mediterranean diet (MD), on the other hand, is characteristic of the European southern area [36] and the aMED score may be a more adequate food model score to study the Portuguese dietary pattern. The MD is distinguished by consuming abundant quantities of vegetables, pulses, fruits, nuts, whole grain cereals, and unsaturated fatty acids, predominantly derived from olive oil. It is also characterized by a reduced consumption of meat and meat products and a regular consumption of fish, while having a moderate intake of dairy products, primarily cheese or yogurt, and moderate amounts of ethanol [37]. 

The MD, rich in antioxidants and anti-inflammatory components, has demonstrated a strong association with lower rates of mortality and morbidity from chronic diseases [38,39,40]. It has been shown to modulate the production of some inflammatory mediators [41] and eNO [42]. Moreover, high adherence to the traditional Mediterranean diet increased the chances of asthma being under control among adults [37]. It has also been observed that there is a protective association between the “fish, vegetables, and fruit” dietary pattern and current asthma (OR = 0.84; 95% CI, 0.73–0.98) and current medicated asthma (OR = 0.84; 95% CI, 0.72–0.98). Additionally, the Mediterranean diet appears to be protective of obesity and obesity-related diseases [40]. This dietary pattern may also be an adequate food model for weight loss and obesity prevention [40]. 

Despite the complex interplay between obesity, diet, and airway inflammation, these three factors are not frequently examined together in research studies. We aimed to further explore the association between the alternate Mediterranean diet (aMED) score, a traditional European dietary pattern, with levels of airway inflammation in school-aged children. In addition, we will consider children’s body mass index (BMI), given its relevant impact on respiratory health [9]. 

## 2. Materials and Methods

### 2.1. Study Design and Participants

**Figure 1:**
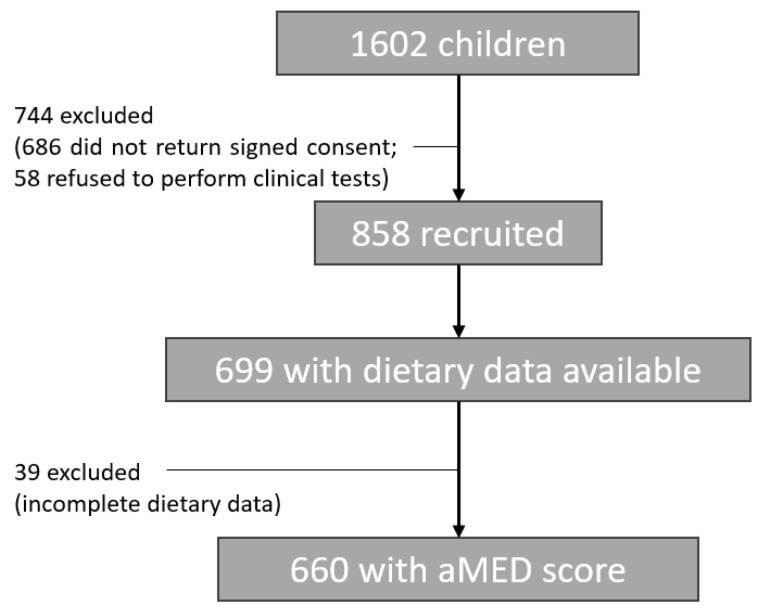
A flow chart of the study recruited and included participants.

This is a cross-sectional study that occurred from 2014 to March 2015. The study involved 1602 school-children (7–12 years old) enrolled in public schools in Porto, Portugal [33]. Of the 858 children providing signed informed consent and agreeing to the clinical procedures, 660 (76.9%) had complete nutritional data regarding to aMED score and were incorporated into the analysis (Figure 1). Written consent was obtained from the legal guardian of each child, and the study was approved by the ethics committee of our University Hospital (ARIA 248-13) and conducted in accordance with the Helsinki Declaration. 

### 2.2. Participants Assessment

#### 2.2.1. Dietary and Diet Quality Assessment

The Mediterranean dietary pattern was evaluated using the aMED score, from the original Mediterranean Diet Scale [43], and based on eight selected food items consumption: fish, red and processed meats, vegetables, pulses, fresh fruits, nuts, whole grains, and the ratio of monounsaturated to saturated fat (MUFA:SFA). The original score includes ethanol (alcohol), but since children do not consume alcohol, it was excluded from the scoring system. For each of the eight components, one or zero points are assigned based on the sex-specific median intake of the study participants. One point was given if the intake was at or above the median, and zero if below the median, except for red and processed meats, in which intake below the median was scored one point. The total aMED score (from zero to eight points) assesses adherence to the Mediterranean diet, where higher scores represent a healthier diet. 

To gather dietary information, a single 24 h food recall questionnaire was administered to the children by a trained interviewer. The questionnaire followed established procedures and included the use of a photograph atlas to assist in estimating portion sizes, asking in-depth questions about the children’s food and beverage intake over the past 24 h, with specific details such as brands and quantities [44]. The nutritional data and total energy intake (in kilocalories) were calculated using the Food Processor^®^ software, SQL®, V3 developed by ESHA Research in the United States. This software incorporates databases containing information on the nutritional composition of Portuguese foods.

#### 2.2.2. Anthropometry

Body weight (in kilograms) was assessed via Tanita™ BC-418 Segmental Body Analyzer (Middlesex, UK), a digital scale and height (in centimeters) recorded using a portable stadiometer. BMI (kg/m^2^) was calculated by dividing weight by the square of height. Participants were classified as non-overweight/non-obese (below the 85th percentile) and overweight/obese (equal to or above the 85th percentile) based on specific sex and age BMI percentiles, from the US Centers for Disease Control and Prevention (CDC) [45].

#### 2.2.3. Airway Inflammation

Fractional eNO was measured with the NObreath analyzer (Bedfont Scientific Ltd., Rochester, Kent, UK). The results were categorized based on the official criteria for children established by the American Thoracic Society (ATS) [46] and registered as parts per billion (ppb). To classify exhaled nitric oxide levels, a cutoff value of 35 ppb or higher was used, indicating elevated levels of fractional eNO [46].

#### 2.2.4. Covariates

The selection of potential confounding factors was determined by a combination of logical reasoning based on conceptual understanding and empirical evidence [47]. Components such as age, sex, atopy, total energy intake, breastfeeding, and tobacco exposure were chosen based on both our understanding of the subject matter and the evidence available in prior research [48,49]. Skin-prick tests (SPT) were carried out on the forearms of children via a QuickTestTM kit. More information on the standard procedures used is described elsewhere [50]. Atopy was considered if a positive SPT (3 mm-diameter wheal) to at least one allergen 15 min after the results were read. Exposure to tobacco at home was confirmed if a positive answer was given to the question “Is your child exposed to tobacco smoke at home?”, and breastfeeding was determined according to the question “Has your child been breastfed?”. Parental education was categorized according to their regular school education in three levels: ≤9 years; ≥10 to ≤12 years; and >12 years [51].

#### 2.2.5. Statistical Analyses 

All statistical analyses were conducted using the SPSS^®^ statistical package software version 27.0. To assess the normality of continuous variables, the skewness and kurtosis test was employed. The participants’ characteristics are expressed as percentages for categorical variables across the entire sample, as median (25th–75th percentile) for continuous variables if non-normally distributed, and as mean ± standard deviation (SD) for normally distributed ones.

To examine differences between children with and without airway inflammation and between non-overweight/non-obese vs. overweight/obese children, independent-sample *t*-tests were used for continuous variables, while chi-squared tests were employed for categorical variables. When variables were non-normally distributed, the Mann–Whitney test was utilized for inferential analysis.

Logistic regression models (odds ratios [OR], 95% confidence intervals [CI]) were employed to estimate the associations between aMED score and airway inflammation. To evaluate the goodness of fit of the logistic regression models the Hosmer–Lemeshow test was conducted.

Significant differences were defined if a α-value had less than 5%, 95% confidence interval (*p* < 0.05).

## 3. Results

The characteristics of the included study participants are presented in Table 1.

The mean age was 8.68 (±0.77) years, 49.1% (*n* = 324) were girls, and 25.6% (*n* = 169) were overweight or obese children. Regarding airway inflammation, 13% (*n* = 86) had elevated levels of eNO (eNO ≥ 35 ppb). No significant differences were found between children without and with airway inflammation except for age (8.66 ± 0.77 y vs. 8.85 ± 0.75 y), obesity/overweight classification [155 (27%) vs. 14 (16.3%)], MUFA/SFA ratio [1.18 (0.92–1.46) vs. 1.11 (0.85–1.33)], and atopy [172 (30.4%) vs. 55 (64.7%)].

Additionally, there were no statistically significant differences between overweight/obese and non-overweight/non-obese children, except for total energy intake (TEI) and parental education. Overweight/obese children had a higher energy intake [2241.10 (1928.69–2519.82) vs. 2135.95 (1848.18–2468.38)] and parents with lower educational levels (42.2% vs. 33.2%) (see Table 2).

After adjusting for age, sex, atopy, breastfeeding, parental education level, tobacco exposure at home, and total energy intake, higher aMED score had no significant association with airway inflammation (OR = 0.84; 95% CI 0.69–1.02). However, non-overweight/non-obese children with higher aMED score had lower odds of having eNO levels greater than or equal to 35 ppb (OR = 0.77; 95% CI 0.61–0.97), as presented on Table 3. 

The Hosmer-Lemeshow test was performed to assess the fit of both logistic regression models, and it indicated a calculated chi-square value of 12.91 with a *p*-value of 0.115 (for the non-overweight/non-obese model regression) and a calculated chi-square value of 5.50 with a *p*-value of 0.704 (for the overweight/obese model regression). At a significance level of 5%, we cannot reject the null hypothesis. Therefore, we can conclude that both models are adequately adjusted. 

## 4. Discussion

This study unveiled that having a better adherence to a Mediterranean diet appears to reduce the odds of having higher airway inflammation in school-aged children but only among those who are non-overweight or non-obese.

Other studies have proposed a beneficial effect of Mediterranean diet on respiratory health [31,37,41,42,52]. Adults with higher adherence to the traditional Mediterranean diet were more likely to have a lower value of eNO levels (OR = 0.22; 95% CI = 0.05–0.85) [37]. Moreover, the Mediterranean diet has been shown to modulate the production of some inflammatory mediators associated with respiratory health such as IL-4 and IL-17 [41] as well as eNO [42]. Accordingly, a randomized controlled trial in asthmatic children showed that a Mediterranean diet supplemented with two meals/week of 150 g of fatty fish for six months, to reduce airway inflammation as measured by eNO (β = −14.15 ppb, 95% CI −27.39; −0.91) comparatively with the usual diet [42]. Another study also suggested an inverse significant relationship between salad intake and eNO in children [53]. 

As previously mentioned, fiber and fats have the ability to influence the gut microbiota, resulting in either an increase or decrease in the production of short-chain fatty acids, respectively [17,18]. Whole grains, along with fruits, legumes, nuts, and vegetables, are essential components of the aMED score [11,54]. These food groups provide vitamins, minerals, and bioactive compounds, and they are also a valuable source of fiber. Halnes et al. assessed the immediate influence of a meal that was rich in soluble fiber, compared to a meal comprising simple carbohydrates. The study focused on asthmatic airway inflammation and explored changes in the expression of genes related to free fatty acid receptors, GPR41 and GPR43. The findings revealed that the group consuming the meal rich in soluble fiber exhibited significantly lower levels of airway inflammation biomarkers, including NO. Furthermore, lower levels of airway inflammation biomarkers were associated with increased expression of sputum genes GPR41 and GPR43 [17]. Systematically increased NO can damage organs and consume antioxidants impeding their protective properties against oxidative stress [55]. It appears that pro-inflammatory cytokines could increase the activity of the iNOS enzyme, promoting NO production [53]. A healthier diet may promote a reduction in pro-inflammatory cytokines through its antioxidant and anti-inflammatory properties [18]. This reduction in pro-inflammatory cytokines may facilitate a decrease in NO production and consequently in airway inflammation. However, a low antioxidant dietary intake, usually reflected by a low consumption of fruits, whole grains, and vegetables, as well as an intake of saturated fat consumption and adhering to a typical obesogenic Western diet, can increase oxidative damage to the airways via the generation of ROS [20]. It is important to recognize that nutrients are not consumed in isolation, and the complex interaction between various components within the food matrix and the overall dietary pattern could contribute to lower airway inflammation levels [56]. The negative associations observed in the current study may be explained by considering the combined and synergistic effects of nutrients and phytochemicals obtained from the Mediterranean diet. 

A higher adherence to the Mediterranean diet in children may be crucial for the development of mechanisms against inflammation [41,42], and although the evidence is limited, it may also be essential for maintaining a healthy body weight [57].

In our study, the protective effect of diet was only observed among children who were non-overweight/non-obese. Similarly, in a study including adults, even though a healthier diet was associated with better respiratory health (lower asthma symptom score), when individuals were stratified according to their BMI, some statistically significant associations were lost [21]. Additionally, it was previously observed by our research team that non-overweight/non-obese children with higher levels of the Healthy Eating Index-2015 score had lower levels of airway inflammation, asthma diagnosed by a physician, and asthma with medication use [35]. We also verified the association between aMED score and the prevalence of asthma, but no significant results were found . The Mediterranean diet is acknowledged for its overall anti-inflammatory effects [41]. Nonetheless, a more adequate dietary pattern for asthma prevention is not fully established, and other components are not present in this score, including trans-saturated fatty acids, the *n*-6:*n*-3 ratio, and dietary diversity, that may be essential when considering asthma prevention [58].

It was previously indicated that obese individuals may be more prone to having higher circulating concentrations of inflammatory mediators [59]. The anti-inflammatory effects of a healthier diet may not be enough to compensate for the negative consequences of having excessive weight, considering that obesity leads to a chronic inflammatory state [59]. Furthermore, although obesity is known to stimulate inflammatory pathways, when researchers investigated the relationship between eNO and obesity in obese children, adolescents, and adults, they did not find a positive association between obesity and eNO [60,61,62,63]. This phenomenon could be due to a mechanical impact of weight on the thoracic region that hinders the generation and diffusion of nitric oxide [64]. Alternatively, obesity may lead to an incongruence between the growth of the lungs and the airways in children [65]. Apart from the mechanical obstructive effect of excessive weight, another reason for low eNO measurement in children with overweight/obesity could be that adiposity leads to neutrophilic systemic inflammation rather than eosinophilic inflammation [63] or to the limited production of NO in the airways and increased NO metabolism [63,66]. An increase in oxidative stress could lead to an elevated production of reactive oxygen species, which could result in the conversion of airway nitric oxide into ROS [60].

Furthermore, while the aMED score incorporates various components of the diet, certain aspects such as the *n*6:*n*3 ratio, trans saturated fat consumption, and vegetable diversity are not taken into account. Overweight/obese children with higher aMED scores may have a lower vegetables diversity intake and a higher *n*-6:*n*-3 ratio and trans saturated fat consumption. These factors have demonstrated to be relevant when studying airway inflammation [33,67,68]. In addition, even though not statistically significant, non-overweight/non-obese children have overall higher median (25th–75th) levels in components that are considered healthy by the aMED score and a lower median level in the component that is not considered to be beneficial for health (red and processed meats), (Table 3).

There are a few limitations in our study that need to be acknowledged. Firstly, as this is a cross-sectional study, reverse causation may occur [69]. Also, the cross-sectional design hinders the establishment of causal relationships between higher adherence to a Mediterranean diet and airway inflammation. Secondly, we used a 24 h recall questionnaire, primarily focused on short-term intake and which did not account for seasonal variations. Since a single day does not accurately reflect typical intake, it is preferable to use multiple recalls to report an individual’s habitual intake [70]. However, we collected comprehensive information regarding portion sizes, ingredients in mixed dishes, and specific brand names of commercial products, allowing for a thorough characterization of consumption and dietary intake quality [70]. Using a 24 h recall questionnaire does not disrupt children’s dietary habits or cause them to modify their eating behaviors due to the time-consuming nature of recording or being aware of their diet being evaluated. This method allows us to estimate their current diet without inducing any alterations in their dietary behaviors [71]. The dietary data collected could be influenced by recall bias and indirect reporting, especially because children’s self-reports of their diet are prone to errors due to limited food knowledge and memory [72]. However, specially trained interviewers obtained the children’s food recall questionnaires, with experience in eliciting information from children without suggesting responses. To address the challenge of estimating portion sizes accurately and minimizing misreporting in dietary consumption, they used food models and photographs to quantify portion sizes [73]. At the group level, the 24 h dietary recall demonstrated good agreement and satisfactory reporting between energy intake and measured total energy expenditure [44]. Additionally, the 24 h recall may be preferable to determine the usual dietary intake in large groups of participants, as it is easier for children to remember their most recent food consumption [74]. Regarding anthropometry, weight classifications were determined using BMI through height and weight, which does not take into account body composition [75]. Body adiposity appears to be a more appropriate indicator of obesity [76,77,78]. Nonetheless, BMI was calculated using measured weight and height, avoiding parental self-perception as children’s overweight/obesity status are usually underestimated by most parents [79].

Our study also has a variety of strengths. To the best of our knowledge, this study is the first one exploring the adherence to the Mediterranean diet with airway inflammation stratifying children based on their BMI. This research involved a large number of individuals, and our research took into account relevant confounders such as atopy, parental education, total energy intake, tobacco exposure, and breastfeeding [47,48]. Nonetheless, residual confounding may still occur. One other strength is that the same research team gathered detailed health data, assuring a relatively unbiased evaluation of outcome prevalence. Moreover, respiratory and dietary outcomes were assessed at the same point in time [80]. Lastly, using the alternate Mediterranean diet score presents several positive aspects due to its holistic approach, which enables us to capture the synergistic effects within the diet. Unlike focusing on individual components or nutrients, the aMED score runs through diverse factors of the dietary pattern. This comprehensive assessment allows for a more accurate representation of the overall diet quality and its impact on health outcomes. Additionally, the MD, despite being more and more abandoned by the Portuguese population in recent times, still presents a lot of characteristics that are typical of Portuguese eating habits [81]. 

In conclusion, this research proposes that in non-overweight/non-obese school-aged children, a healthier diet is associated with lower levels of eNO. This work highlights the relevance of promoting a healthy diet that includes a variety of nutrient-rich components, such as vegetables, fruits, whole grains, fish, healthy fats, and healthy sources of protein, regarding airway inflammation. Understanding the potential impact of food consumption on airway inflammation and its interplay with obesity can provide valuable insights for developing clinical guidelines and public health recommendations for improving respiratory health. However, there are still significant gaps in comprehending the specific foods or dietary patterns that individuals should integrate to enhance their respiratory health, particularly regarding airway inflammation.

## Figures and Tables

**Table 1:** Summary of the study participant’s characteristics.

	Total, *n* = 660 (100%)	Fractional eNO < 35 ppb, *n* = 574 (87%)	Fractional eNO ≥ 35 ppb, *n* = 86 (13%)	*p*-Value
Age (years), mean ± SD	8.68 ± 0.77	8.66 ± 0.77	8.85 ± 0.75	0.041
Sex, female, *n* (%)	324 (49.1%)	288 (50.2%)	36 (41.9%)	0.15
BMI				0.034
Non-overweight/non-obese (*p* < 85th)	491 (74.4%)	419 (73%)	72 (87.7%)	
Overweight/obese (*p* ≥ 85th)	169 (25.6%)	155 (27%)	14 (16.3%)	
aMED score, mean ± SD	2.76 ± 1.48	2.80 ± 1.46	2.52 ± 1.57	0.11
Vegetables (g), median (25th–75th)	87.12 (34.27–150.77)	87.12 (29.54–149.14)	87.12 (45.04–165.70)	0.92
Fruits (g), median (25th–75th)	174.0 (84.0–303.5)	174.0 (84.0–302.0)	174.0 (74.00–305.75)	0.48
Ratio MUFA/SFA	1.17 (0.92–1.45)	1.18 (0.92–1.46)	1.11 (0.85–1.33)	0.050
Fish (g), median (25th–75th)	0.00 (0.00–100.00)	0.00 (0.00–100.00)	0.00 (0.00–62.50)	0.19
Nuts (g), median (25th–75th)	0.00 (0.00–0.00)	0.00 (0.00–0.00)	0.00 (0.00–0.00)	0.37
Whole grains (g), median (25th–75th)	0.00 (0.00–0.00)	0.00 (0.00–0.00)	0.00 (0.00–0.00)	0.35
Legumes (g), median (25th–75th)	0.00 (0.00–0.00)	0.00 (0.00–9.76)	0.00 (0.00–0.00)	0.13
Red and processed meat (g), median (25th–75th)	74.8 (20.0–140.0)	72.65 (20.0–140.0)	84.85 (20.0–138.83)	0.65
Total energy intake (kcal), median (25th–75th)	2164.75 (1867.95; 2476.06)	2176.93 (1872.69–2492.47)	2081.45 (1778.45–2436.30)	0.19
Breastfeeding ^a^, *n* (%)	474 (71.8%)	350 (71.3%)	124 (73.4%)	0.28
Tobacco exposure ^b^, *n* (%)	144 (24.0%)	127 (24.2%)	17 (22.4%)	0.73
Atopy ^c^, *n* (%)	227 (34.9%)	172 (30.4%)	55 (64.7%)	0.001
Parental education ^d^, *n* (%)				0.73
<9 years	188 (28.5%)	161 (28%)	27 (31.4%)	
10–12 years	161 (24.4%)	141 (24.6%)	20 (23.3%)	
>12 years	180 (27.3%)	159 (27.7%)	21 (24.4%)	

Note: Abbreviations: aMED: alternate Mediterranean score. FeNO: fractional exhaled nitric oxide. MUFA: monounsaturated fatty acids; %TEI: total energy intake; SFA: saturated fatty acids. ^a^ child was breastfed; ^b^ child exposed to tobacco smoke at home; ^c^ positive skin-prick test; ^d^ number of successfully completed years of formal schooling. To examine differences between children with and without airway inflammation independent-sample *t*-tests were used for continuous variables, while chi-squared tests were employed for categorical variables. When variables were non-normally distributed, the Mann–Whitney test was utilized for analysis.

**Table 2:** Summary of participant’s characteristics according to BMI status.

	Total, *n* = 660 (100%)	Non-Overweight/Non-Obese, *n* = 491 (74.4%)	Overweight/Obese, *n* = 169 (25.6%)	*p*-Value
Age (years), mean ± SD	8.68 ± 0.77	8.66 ± 0.76	8.76 ± 0.81	0.15
Sex, female, *n* (%)	324 (49.1%)	242 (49.3%)	82 (48.5%)	0.86
aMED score, mean ± SD	2.76 ± 1.48	2.79 ± 1.51	2.69 ± 1.39	0.44
Vegetables (g), median (25th–75th)	87.12 (34.27–150.77)	87.12 (44.04–153.60)	87.12 (29.07–149.21)	0.37
Fruits (g), median (25th–75th)	174.0 (84.0–303.5)	174.0 (84.00–308.00)	174.0 (80.0–300.0)	0.55
Ratio MUFA/SFA	1.17 (0.92–1.45)	1.18 (0.92–1.47)	1.14 (0.92–1.43)	0.89
Fish (g), median (25th–75th)	0.00 (0.00–100.00)	0.00 (0.00–100.00)	0.00 (0.00–100.00)	0.77
Nuts (g), median (25th–75th)	0.00 (0.00–0.00)	0.00 (0.00–0.00)	0.00 (0.00–0.00)	0.43
Whole grains (g), median (25th–75th)	0.00 (0.00–0.00)	0.00 (0.00–0.00)	0.00 (0.00–0.00)	0.69
Legumes (g), median (25th–75th)	0.00 (0.00–0.00)	0.00 (0.00–0.00)	0.00 (0.00–9.84)	0.74
Red and processed meat (g), median (25th–75th)	74.8 (20.0–140.0)	73.20 (20.00–141.00)	76.00 (20.00–140.00)	0.58
Total energy intake (kcal), median (25th–75th)	2164.75 (1867.95; 2476.06)	2135.95 (1848.18–2468.38)	2241.10 (1928.69–2519.82)	0.049
Breastfeeding ^a^, *n* (%)	474 (71.8%)	350 (81.4%)	124 (83.2%)	0.62
Tobacco exposure ^b^, *n* (%)	144 (24.0%)	102 (22.8%)	42 (27.5%)	0.24
Atopy ^c^, *n* (%)	227 (34.9%)	173 (35.7%)	54 (32.3%)	0.43
Parental education ^d^, *n* (%)				0.021
<9 years	188 (28.5%)	131 (33.2%)	57 (42.2%)	
10–12 years	161 (24.4%)	116 (29.4%)	45 (33.3%)	
>12 years	180 (27.3%)	147 (37.3%)	33 (24.4%)	

Note: Abbreviations: aMED: alternate Mediterranean score. FeNO: fractional exhaled nitric oxide. MUFA: monounsaturated fatty acids; SFA: saturated fatty acids; %TEI: total energy intake. ^a^ child was breastfed; ^b^ child exposed to tobacco smoke at home; ^c^ positive skin-prick test; ^d^ number of successfully completed years of formal schooling. To examine differences between non-overweight/non-obese vs. overweight/obese children, independent-sample *t*-tests were used for continuous variables, while chi-squared tests were employed for categorical variables. When variables were non-normally distributed, the Mann–Whitney test was utilized for inferential analysis.

**Table 3:** Analysis of the association between the aMED score with airway inflammation.

	aMED Score Crude Model, OR (95% CI)	*p*-Value	aMED Score Adjusted Model, OR (95% CI)	*p*-Value
Increased Levels of Exhaled Nitric Oxide (≥35 ppb)				
All participants	0.88 (0.75–1.03)	0.109	0.84 (0.69–1.02)	0.08
Non-overweight/non-obese	0.85 (0.72–1.00)	0.055	0.77 (0.61–0.97)	0.025
Overweight/obese	1.06 (0.71–1.57)	0.779	1.57 (0.88–2.79)	0.13

Note: Abbreviations: aOR: adjusted odds ratio; aMED: alternate Mediterranean score; FeNO: fractional exhaled nitric oxide. Logistic regressions were adjusted to age, sex, parental education, atopy, breastfeeding, tobacco exposure at home, and total energy intake. Significant differences were defined with an α-value of less than 5%, 95% confidence interval, (*p* < 0.05). Logistic regression models were employed to estimate the associations between aMED score, and airway inflammation.

## Data Availability

The data that support the findings of this study will be made available by the authors upon reasonable request.
